# Delays in diagnosis and treatment initiation for congenital cytomegalovirus infection - Why we need universal screening

**DOI:** 10.3389/fped.2022.988039

**Published:** 2022-09-14

**Authors:** Styliani Alifieraki, Helen Payne, Chantal Hathaway, Rachel Wei Ying Tan, Hermione Lyall

**Affiliations:** ^1^St Mary's Hospital, Pediatric Infectious Diseases, Imperial College Healthcare NHS Trust, London, United Kingdom; ^2^School of Medicine, Imperial College London, London, United Kingdom

**Keywords:** congenital cytomegalovirus, infants, neurodevelopmental impairment, hearing loss, delays, universal screening

## Abstract

**Introduction:**

Congenital cytomegalovirus (cCMV) is the leading cause of neurodevelopmental and hearing impairment from *in-utero* infection. Late diagnosis results in limited treatment options and may compromise long-term outcome.

**Methods:**

A retrospective audit of infants with cCMV referred to a Tertiary Pediatric Infectious Diseases center from 2012–2021. Data collected included timing of diagnostics, treatment initiation and reasons for delays.

**Results:**

90 infants with confirmed cCMV were included, 46/90 (51%) were symptomatic at birth. Most common reasons for diagnostics in asymptomatic infants were failed newborn hearing screening (17/44, 39%) and antenatal risk-factors (14/44, 32%). Median age at cCMV diagnosis was 3 (range 0–68) and 7 (0–515) days, with median referral age 10 (1–120) and 22 (2–760) days for symptomatic and asymptomatic infants respectively. There was a significant risk of delay in diagnosis (>21 days) for asymptomatic infants [RR 2.93 (1.15–7.45); *p* = 0.02]. Of asymptomatic infants who received treatment, 13/24 (54%) commenced it within 28 days of life, a significant delay in treatment compared to 30/36 (83%) symptomatic infants [RR 2.75 (1.18–6.43); *p* = 0.02]. The commonest reason for delayed treatment initiation was delayed first diagnostic test for both symptomatic 4/6 (67%) and asymptomatic infants 9/11 (82%).

**Conclusions:**

Delays in diagnosis and treatment for cCMV are unacceptably frequent and significantly higher in asymptomatic infants. Our study highlights the need for increased awareness among healthcare professionals, reconsideration of age-targets for Newborn Hearing Screening, and research that addresses the barriers to implementation of universal screening, which would ultimately facilitate prompt diagnosis and management of all infants with cCMV.

## Introduction

Cytomegalovirus (CMV) is the leading cause of neurodevelopmental and hearing impairment from congenital infection (1) with substantial clinical and economic burden (2–7). The estimated birth prevalence of congenital CMV (cCMV) is 0.67% worldwide with a three-fold increase in low- and middle-income countries (8), greater numbers than better-known conditions such as Down syndrome or spina bifida (9).

“Asymptomatic” infants with cCMV are those without manifestation of symptoms of cCMV at birth, and these make up 85–90% of all infants with cCMV (10). Current treatment guidelines are based on randomized controlled trial data (11, 12) and recommend commencing treatment with valganciclovir within 28 days of life in infants with symptomatic cCMV and continuing this treatment for 6 months. The trial demonstrated that early treatment with valganciclovir results in CMV virological control, reduced rates of hearing impairment and improved neurodevelopmental scores (11, 12). However, without universal screening it is estimated that <25% of children are diagnosed due to variable, non-specific and delayed clinical manifestations (1, 3, 9, 13–20). Limitations to clinical diagnosis arise from the variable presentation of clinical signs and symptoms of the disease; signs that may be subtle or non-specific and potentially attributable to numerous other conditions e.g., intrauterine growth restriction (IUGR), petechiae, anemia, hepatosplenomegaly, jaundice and microcephaly. In the UK alone, this amounts to approximately 420 delayed or missed diagnoses of symptomatic cCMV that could have benefitted from therapeutic intervention (21). Studies suggest that up to 13.5% of asymptomatic infants will develop late-onset neurodevelopmental impairment or hearing loss (22) and without a universal screening programme, these infants are not detected in time to receive treatment that might prevent these late-onset sequelae. In the UK, this potentially amounts to a further 425 infants that might have benefitted from treatment – and globally this, alongside missed diagnoses of symptomatic cCMV, reflects an estimated 0.14% of all births, or 196,000 infants, that may have missed the opportunity to be treated for cCMV (23).

cCMV infection is the primary cause of non-genetic sensorineural hearing loss (SNHL) accounting for 10–20% of all children with hearing impairment (24). 33–71% of infants with symptomatic cCMV and 0–15% of asymptomatic infants develop late-onset SNHL, with 8–62% and 3–18% manifesting as late-onset disease respectively (7, 10, 13, 25–33). For this reason, 6-monthly audiological monitoring is recommended for all infants with cCMV regardless of “symptom-status.” Vestibular function may also be variably affected in children with cCMV, even among those with normal hearing, and vestibular assessment should be part of the follow-up too (34).

Adverse neurodevelopmental outcomes are another leading complication of cCMV that require monitoring and includes cognitive impairment (66 vs. 6.5%), visual impairment (22 vs. 2.4%) and motor impairment (19 vs. 10%) in symptomatic vs. asymptomatic infants, respectively (10, 15, 26–30, 35–37). This highlights the importance of prompt diagnosis, timely treatment initiation and follow-up of all infants diagnosed with cCMV for early recognition and management of the long-term sequalae (7, 38).

The aim of our study was to describe the timing of diagnosis and treatment initiation of all infants diagnosed with cCMV at our tertiary Pediatric Infectious Diseases center, and to understand the reasons for these delays.

## Materials and methods

### Study population

This study is a retrospective audit of all infants with cCMV referred to our Tertiary Pediatric Infectious disease center, St Mary's Hospital, Imperial College NHS Healthcare Trust (ICHT) London, from April 2012 to July 2021. All infants referred to our Pediatric Infectious Disease Center with suspected cCMV during this period were screened for eligibility. We included all infants who were diagnosed with cCMV based on either two positive CMV PCRs on bodily secretions (urine, saliva, blood) within the first 3 weeks of life; or one CMV positive PCR on bodily secretions or a positive CMV PCR on the Guthrie card testing plus clinical features in keeping with a diagnosis of cCMV (as described below). Infants were excluded if death occurred in the neonatal period. Children aged 2 years and above at the time of retrospective diagnosis of cCMV, typically through Guthrie card testing following diagnosis of sensorineural hearing loss, were also excluded.

Infants were grouped into symptomatic or asymptomatic and analyzed separately. Symptomatic was defined as infants with clinical manifestations of cCMV, which could reasonably be detected *via* the newborn infant physical examination (2, 39). These manifestations included microcephaly (occipitofrontal circumference >2 standard deviations below the mean for a given age, sex, and gestation), IUGR (fetal growth deceleration and/or neonates born with clinical features of intrauterine malnourishment in relation to their gestational age), hepatomegaly, splenomegaly, petechiae or purpura. Infants with laboratory findings consistent with cCMV identified prior to initial discharge from hospital following birth were also considered symptomatic. The laboratory findings included anemia, thrombocytopenia, prolonged jaundice with transaminasaemia and conjugated hyperbilirubinaemia. Those with sensorineural hearing loss detected on repeat audiological examination and with no other clinical manifestations were included in the asymptomatic group (39).

Sequelae of cCMV infection including seizures of any age, progressive hearing loss, developmental delay, language delay, and learning difficulties was collected. Progressive hearing loss was defined as a deterioration in hearing on follow up assessment by Audiology Specialists after 6 months of age. All children with confirmed cCMV undergo routine developmental assessment as part of follow up and are referred for formal developmental assessment by the Community Pediatric service at 1 year. Bayley III is the recommended tool for developmental assessment, however the tools used in practice varied between the Community centers. Developmental delay was defined as delay in at least two fields of development (gross motor, fine motor, speech and language and social interactive skills) after the age of one. Language delay was defined as those children identified as having a language delay by the Speech and Language specialists after the age of 2 years. Learning difficulties included any child over the age of five identified as having additional educational needs by the Community Pediatrician or Educational psychologist.

### Data collection

Data was collected from the electronic patient records including referral letters, clinic letters and laboratory reports. Data included demographics (sex, gestational age at birth, birth weight), reason for suspecting cCMV (e.g., IUGR, failed newborn hearing screen), diagnostic process (e.g., test indication, sample type, day of diagnosis and referral), treatment received and any sequalae of infection.

### Audit standards

Our audit standard was set that the decision for treatment-initiation for cCMV should be made by the 28^th^ day of life, as per the European expert consensus (39). Indications for treatment included significant single-organ disease or multi-organ involvement and any central nervous system (CNS) disease. Infants with isolated sensorineural hearing loss were also considered for treatment. Patients with no symptoms or mild disease, such as isolated IUGR, hepatomegaly with normal liver enzymes, mild and transient thrombocytopenia or isolated elevation of liver enzymes were not eligible for treatment. This study aimed to determine the proportion of infants who attained that standard and identify the reasons if they did not. Each step in the diagnostic and referral process was considered and acceptable timings were defined as follows: first test completed prior to 21^st^ day of life or on day of failed hearing screen, test results received within seven days of testing, referral to tertiary team received within seven days of receiving test result, appointment with Infectious diseases team within seven days of receiving the referral. Infants were excluded if no data was available for time of first test, age at referral and age at initiation of treatment. If age at referral was unknown, age at first consultation with the service was used.

### Analysis

Median timings for all steps in the diagnostic and referral process were calculated using excel. Fisher exact probability test was used to compare the timing of first diagnostic test and the initiation of treatment between the asymptomatic and symptomatic group. Risk ratios (RR) and 95% confidence intervals (CI) were calculated. Statistical significance was set at two-tailed *p* < 0.05.

## Results

One hundred and fifty-five infants with potential CCMV were referred to our Pediatric Infectious Disease (PID) department between April 2012 and July 2021, of which 90 infants with confirmed CCMV were included in this study. The demographic profile of the patients is reflected in Table 1. There were 64 term infants (64/86, 74.4%) with the median gestation of 38 weeks (Interquartile range (IQR) 37–40, range 37–42), and 23 preterm infants with median gestation of 35 weeks (IQR 30.5–36, range 27–36). The median birth weight was 2640 g (range 925–4100g). There were 26 infants (26/80, 32.5%) who had IUGR defined as a fetal growth deceleration and/or neonates born with clinical features of intrauterine malnourishment in relation to their gestational age.

**Table 1:** Demographics of infants with cCMV in this population.

	**Symptomatic No. (%)**	**Asymptomatic No. (%)**
Total no:	46 (51)	44 (49)
**Gender**		
Male	20 (43.5)	22 (50)
Female	26 (56.5)	22 (50)
**Gestation**		
Term	29 (65.9)	34 (81.0)
Mean (range)	36.3 (27–41)	38.2 (28–42)
Median [IQR]	37 [36–38]	39 [37–40]
**Birth weight (g)**		
IUGR	26 (60.5)	0 (0)
Mean (range)	2,352.4 (925–4,100)	3,126.9 (2,080–4,050)
Median [IQR]	2,380 [2,055–2,630]	3,094 [2,774–3,525]

IUGR, intra-uterine growth restriction; IQR, Interquartile range.

Symptoms were detected from abnormal physical examination and laboratory results in 46/90 infants (51%), who were considered symptomatic at diagnosis (46/90, 51%). The most common symptoms were thrombocytopenia (22/46, 47.8%), and IUGR (26/46, 56.5%, Table 2). Among asymptomatic infants, diagnostic tests were mostly performed due to a failed newborn hearing screening (17/44, 39%), and antenatal suspicion (14/44, 32%, Table 2).

**Table 2:** Reasons for testing for CMV.

**Clinical symptoms or abnormal laboratory tests in symptomatic infants**	**No. (%)**	**Reasons for testing in asymptomatic infants**	**No. (%)**
IUGR	26 (56.5)	Failed NBHS	17 (39)
Thrombocytopenia	22 (47.8)	Antenatal suspicion	14 (32)
Microcephaly	15 (32.6)	Incidental finding	6 (14)
Petechial rash	11 (23.9)	LBW (<2^nd^ centile)	3 (7)
Hyperbilirubinaemia	8 (17.4)	Postnatal hearing loss	2 (5)
Hepatic symptoms (hepatitis, hepatomegaly)	5 (10.9)	Unknown	2 (5)
Chorioretinitis	3 (6.5)		
Neutropenia	1 (2.2)		

NBHS, Newborn hearing screening; LBW, Low birth weight; IUGR, intrauterine growth restriction. Incidental finding: positive cCMV on urine, saliva or cord blood sample.

The median age of the patients when cCMV diagnosis was made was 3 days (IQR 1–5.5, range 0–68) for the symptomatic patients, and 7 days (IQR 2.25–29.5, range 0–515) for the asymptomatic patients (Table 3). There was a significant delay in reaching diagnosis defined as beyond 21 days of life for the asymptomatic group [RR 2.93 (1.15–7.45); *p*–value 0.02] compared to the symptomatic group. The median time to referral was 10 days in the symptomatic group (IQR 6.5–27.5, range 1–120 days), compared to 22 days in the asymptomatic group (IQR 9.5–38, range 2–720 days) (Table 3).

**Table 3:** Timings of diagnostic test, referral and treatment initiation and reasons for delayed treatment initiation.

	**Symptomatic (*n* = 46)**	**Asymptomatic (*n* = 44)**
	**No. (%) or Median [IQR] (range)**	**No. (%) or Median [IQR] (range)**
**Timing of diagnostic test, referral and treatment initiation**		
Time of first test (day of life)	3 [1–5.5] (0–68)	7 [2.25–29.5] (0–515)
First test <21 days	41 (89.1%)	30 (68.2%)
Time of referral to PID	10 [6.5–27.5] (1–120)	22 [9.5–38] (2–760)
Number treated	36 (78%)	24 (54.5%)
Treatment started	8 [4–16] (1–120)	28 [20.75–67] (6–112)
Treatment started <28 days	30 (83.3%)	13 (54.2%)
	**Symptomatic** **(*****n*** = **6)** **No. (%)**	**Asymptomatic** **(*****n*** = **11)** **No. (%)**
**Reasons for delayed initiation of treatment**		
First testing	4 (67%)	9* (82%)
Receiving results	3 (50%)	3 (27%)
Referral to PID	1 (17%)	2 (18%)
Parental hesitance	0 (0%)	3 (27%)
Unknown	1 (17%)	1 (9%)
Sample errors	0 (0%)	2 (18%)
Clinical uncertainty	1 (17%)	0 (0%)
MRI	0 (0%)	1 (9%)
Audiology	0 (0%)	1 (9%)
PID appointment	0 (0%)	0 (0%)

PID, Pediatric Infectious Diseases team; IQR, Interquartile range; MRI, magnetic resonance imaging. *In one case first testing was delayed due to the time of first audiological assessment.

Of the patients who were symptomatic, 78% met the criteria for treatment (*n* = 36/46, 78%), including 15 with SNHL. Of the 10 symptomatic infants who did not receive treatment, 9 were mildly symptomatic (small for gestational age) with no end-organ disease and 1 did not receive treatment due to parental choice. Just over half of the patients who were asymptomatic at initial presentation met the criteria for treatment (*n* = 24/44, 54.4%). Fifteen infants from the asymptomatic group were treated due to SNHL of whom 8 also had neuroimaging consistent with cCMV, and another 8 had neuroimaging suggestive of cCMV disease. One infant was treated in view of co-infection with HIV. Of the 20 asymptomatic infants that were not treated, 16 had no evidence of end-organ disease, hence no treatment was offered. Three infants were referred following diagnosis of SNHL and were diagnosed with cCMV at more than 1 year of age, and one infant with SNHL was offered treatment but parents declined.

Of the symptomatic infants that received antiviral treatment, 30/36 (83%) started within 28 days of life, compared to 13/24 (54%) of asymptomatic infants. Initiation of treatment was earlier for symptomatic (median day 8, IQR 4–16, range 1–120), compared to asymptomatic infants (median day 28, IQR 20.8–67, range 6–112). Delay in treatment initiation (>28 days) was significant among asymptomatic infants [RR 2.75 (1.18–6.43); *p*–value 0.02] compared to symptomatic infants (Table 3).

There were multiple reasons for delayed treatment initiation (Table 3). The commonest reason was delay in first diagnostic test for both symptomatic 4/6 (67%) and asymptomatic infants 9/11 (82%, Table 4). Other reasons included delay in receiving results in 3/6 (50%) of symptomatic infants, and in 3/11 (27%) of asymptomatic infants. Among the asymptomatic group, other reasons included parental hesitance (3/11, 27%), delayed PID referral (2/11, 18%) and sample errors i.e., sample insufficient or not processed (2/11, 18%).

**Table 4:** Sequelae of infants with cCMV.

**Sequelae**	**Symptomatic (*n* = 46)**	**Asymptomatic *(n* = 44)**
	**No. /available data (%)**	**No. /available data (%)**
Seizure	6/44 (13.6)	1/43 (2.3)
Progressive hearing loss (>6 months old)	5/39 (12.8)	6/40 (15)
Developmental delay (>1 year old)	17/37 (45.9)	5/36 (13.9)
Language delay (>2 year old)	21/34 (61.8)	17/32 (53.1)
Learning difficulties (> 5 year old)	11/21 (52.4)	6/18 (33.3)

The sequelae of cCMV infection are reported in Table 4. 39/90 (43%) of the study population were followed up for more than 5 years.

## Discussion

Our study demonstrates that almost half (46%) of infants with asymptomatic cCMV, and 17% of symptomatic infants do not commence treatment with valganciclovir within the recommended timeframe of 28 days. Although multifactorial, the commonest reason for delay in treatment initiation was delay in first diagnostic test for both symptomatic and asymptomatic infants. Such delays are not acceptable when randomized controlled trial evidence supports early treatment initiation to reduce risk of SNHL and neurodevelopmental impairment, at least in symptomatic infants (11, 12).

This study captures 9 years of experience at a tertiary Pediatric Infectious Disease service in North-West London. Although conducted at a local level, these results are likely to reflect national practice in the UK. Despite the existence of local guidelines for the diagnosis and management of cCMV and the screening of infants who fail the audiological assessment, unacceptable delays occur and impact upon the timely management and follow-up of these infants. Our data does not capture all potential missed diagnoses of cCMV, and it is important to note that diagnosis of cCMV itself is compromised by late presentation and delays in diagnostic investigations due to the requirement, for CMV DNA detection within 21 days of life. The retrieval of the Guthrie card can help with retrospective diagnosis using newborn dried blood spot analysis but is limited by the long turnaround time and maximum sensitivity of approximately 80% due to the test and the fact that not all infants with cCMV are viraemic (40–42).

The multiple factors that contribute to delayed diagnosis and initiation of treatment are each critical components that need to be addressed individually. The leading causes include time of formal audiological assessment, virological laboratory results turnaround, access to investigations, and timely appointments to give parents adequate time to reflect upon treatment decisions. Potential strategies for initiating earlier screening might include targeted screening of high-risk infants e.g., premature infants <30 weeks gestation or with IUGR; performing a CMV saliva test for each newborn hearing test failed in hospital before being referred for formal audiological testing; or reducing the 4-week national Newborn Hearing Screening Programme target of referral for audiological assessment (43) to within 2 weeks. Possible recommendations to address the laboratory turnaround issues might include being able to offer a point-of-care CMV saliva test; or centralize CMV laboratory testing to centers that can offer rapid turnaround and delivery of results. Underpinning both these themes is the absolute requirement to improve awareness amongst the public, and relevant healthcare professionals involved in this pathway: Midwives, Obstetricians, Neonatologists, Pediatricians, Audiologists and Virologists. Ultimately, the ideal approach would be to offer universal screening as part of early infant screening to detect and treat early infection and thereby, where possible, prevent SNHL and neurodevelopmental impairment (11, 12). Early infant screening diagnostics that could be utilized include saliva PCR or high sensitivity blood PCR (44, 45).

Many of the criteria required for a population screening programme are already fulfilled for cCMV: the importance of the problem including frequency and severity; being able to identify a pre-symptomatic or asymptomatic stage; having a test that is largely acceptable to the general population; and knowledge of the condition's natural history (1, 9, 19, 24). The benefits of newborn screening for congenital CMV are multiple: timely diagnosis and initiation of therapy in the majority of children who may otherwise be missed; parental confidence in the diagnosis not being of genetic cause, thereby impacting future decisions for family-planning; and overall reduction in parental stress and anxiety caused by uncertain diagnoses (46). Universal screening is also likely to be cost-effective if total healthcare and societal costs, including loss of productivity, of the burden of cCMV are taken into account (4, 18, 20, 47, 48). Potential negative impacts from screening include possible temporary increased parental stress or altered parent-child relationships from a false positive result; the added costs from unnecessary medical visits or investigations, although this is arguably less than the medical and societal costs of caring for those affected by cCMV (49).

The UK's National Screening Committee's most recent review in March 2022 concluded that it did not recommend screening for cCMV for the following reasons: uncertainty about the best test to use to identify those that will have long-term sequelae; the lack of evidence that early treatment following screening leads to better outcomes than later treatment after symptoms; and how to manage asymptomatic infants (50). Clearly these are all important areas that require further research. As a minimum asymptomatic infants would be monitored for late-onset hearing loss and early intervention, but it may be plausible that some of these infants could benefit from antiviral treatment (51), and trial universal screening programmes could facilitate randomized controlled studies to answer this important question. Our cohort of infants considered asymptomatic at presentation have a rate of sequelae that is generally higher than reported in the literature (5, 15). This might be due to the retrospective design of this study or lack of standardized approaches to neurodevelopmental assessments, however it is also likely to reflect the introduction of targeted CMV screening in infants that fail audiology assessments, thereby increasing the detection of cases of CMV-associated SNHL, which are considered asymptomatic. These sequelae data in asymptomatic infants continues to support the need for universal screening. Notably recent studies have shown that up to 50% of infants with asymptomatic cCMV have abnormal MRI brains in infancy (44, 52–54), which might indicate a subgroup of infants who may benefit from therapeutic intervention.

The timing of initiation of antiviral therapy before 1 month of age is based on studies in symptomatic infants (11, 12), likely infected in early pregnancy, however it is important to reassess the evidence on timing of antiviral initiation as new data evolves. For example, a small recent Japanese study in symptomatic infants with high viral loads demonstrated no difference in SNHL outcomes up to 6 months follow-up between initiation of valganciclovir at <1 month, compared to 1–2 months of age (55). Data is limited on treatment of asymptomatic infants, likely to have been infected during late pregnancy, but in whom the risk of late-onset complications exists (25). It is important to determine if even earlier virological control during infant immunological immaturity, such as within days of birth, might reduce late-onset audiological and neurodevelopmental outcomes, particularly in asymptomatic infants, and if confirmed this would most certainly also endorse early universal screening.

Our study describes and quantifies sizeable delays in diagnosis and treatment initiation for infants with cCMV - delays that need to be addressed to expedite diagnosis and offer treatment in a timely manner. Our findings highlight significantly longer delays in diagnosis and treatment initiation in infants with so-called asymptomatic disease compared to symptomatic, thereby supporting the need for universal screening. Further research is needed to determine which infants within the spectrum of symptomatic to asymptomatic will benefit most from treatment to mitigate late-onset disease. Importantly this dichotomous historic categorization of symptomatic or asymptomatic cCMV, does not take account of MRI brain imaging, which may also have prognostic significance. In future, universal screening at birth, and a points-based neonatal scoring system using symptoms and signs of cCMV will help us to better understand which children need treatment and follow up, and should also reduce the systematic delays, highlighted in this audit of care.

## Data availability statement

The raw data supporting the conclusions of this article will be made available by the authors, without undue reservation.

## Author contributions

HL conceptualized the study. SA, CH, and RT collected the data. All authors analyzed the data and contributed to the writing of the manuscript.

## Conflict of interest

The authors declare that the research was conducted in the absence of any commercial or financial relationships that could be construed as a potential conflict of interest.

## Publisher's note

All claims expressed in this article are solely those of the authors and do not necessarily represent those of their affiliated organizations, or those of the publisher, the editors and the reviewers. Any product that may be evaluated in this article, or claim that may be made by its manufacturer, is not guaranteed or endorsed by the publisher.
